# Antimicrobial susceptibility of *Clostridium difficile* isolated in Thailand

**DOI:** 10.1186/s13756-017-0214-z

**Published:** 2017-06-08

**Authors:** Papanin Putsathit, Monthira Maneerattanaporn, Pipat Piewngam, Daniel R. Knight, Pattarachai Kiratisin, Thomas V. Riley

**Affiliations:** 1 Microbiology & Immunology, School of Pathology & Laboratory Medicine, The University of Western Australia, Crawley, WA 6008 Australia; 2 Department of Medicine, Faculty of Medicine Siriraj Hospital, Mahidol University, Bangkok, 10700 Thailand; 3 Department of Microbiology, Faculty of Medicine Siriraj Hospital, Mahidol University, Bangkok, 10700 Thailand; 4 Department of Microbiology, PathWest Laboratory Medicine WA, Queen Elizabeth II Medical Centre, Nedlands, WA 6009 Australia

**Keywords:** *Clostridium difficile*, Thailand, Antimicrobial susceptibility

## Abstract

**Background:**

Exposure to antimicrobials is the major risk factor associated with *Clostridium difficile* infection (CDI). Paradoxically, treatment of CDI with antimicrobials remains the preferred option. To date, only three studies have investigated the antimicrobial susceptibility of *C. difficile* from Thailand*,* two of which were published in the 1990s. This study aimed to investigate the contemporary antibiotic susceptibility of *C. difficile* isolated from patients in Thailand.

**Methods:**

A collection of 105 *C. difficile* isolated from inpatients admitted at Siriraj Hospital in Bangkok in 2015 was tested for their susceptibility to nine antimicrobials via an agar incorporation method.

**Results:**

All isolates were susceptible to vancomycin, metronidazole, amoxicillin/clavulanate and meropenem. Resistance to clindamycin, erythromycin and moxifloxacin was observed in 73.3%, 35.2% and 21.0% of the isolates, respectively. The in vitro activity of fidaxomicin (MIC_50_/MIC_90_ 0.06/0.25 mg/L) was superior to first-line therapies vancomycin (MIC_50_/MIC_90_ 1/2 mg/L) and metronidazole (MIC_50_/MIC_90_ 0.25/0.25 mg/L). Rifaximin exhibited potent activity against 85.7% of the isolates (MIC ≤0.03 mg/L), and its MIC_50_ (0.015 mg/L) was the lowest among all antimicrobials tested. The prevalence of multi-drug resistant *C. difficile*, defined by resistance to ≥3 antimicrobials, was 21.9% (23/105).

**Conclusions:**

A high level of resistance against multiple classes of antimicrobial was observed, emphasising the need for enhanced antimicrobial stewardship and educational programmes to effectively disseminate information regarding *C. difficile* awareness and appropriate use of antimicrobials to healthcare workers and the general public.

## Background

Antimicrobial exposure is the major risk factor for *Clostridium difficile* infection (CDI) [1]. In Thailand, many antimicrobials are traded as over-the-counter drugs. When coupled with a general lack of knowledge regarding the appropriate use of antimicrobials in the community, misuse is inevitable [2]. In 2007, an Antimicrobial Smart Use programme was introduced in Thailand by the World Health Organisation (WHO) to promote the rational use of antimicrobials in patients with upper respiratory tract infections, simple wounds and acute diarrhoea. The programme aimed to produce a sustainable behavioural change in prescribing practices. The WHO reported a successful pilot intervention and emphasised the importance of collaborative effort and commitment from healthcare practitioners and policy makers in scaling up the programme [2].

Paradoxically, treatment of CDI with antimicrobials remains the preferred option, with vancomycin and metronidazole as first-line therapy. Current knowledge regarding the antimicrobial susceptibility of Thai *C. difficile* isolates is limited to two studies published in the 1990s, and one published in 2015. Among 28 *C. difficile* investigated in 1994, Kusom et al. reported a high level of resistance (≥50%) against cefazolin, cefoperazone, tetracycline, erythromycin, clindamycin, ampicillin, bacitracin and cefoxitin. All but one of the test isolates were resistant to ≥3 antimicrobials [3]. In 1996, Wongwanich et al. investigated 38 *C. difficile* isolates using E-strips and reported the MIC_50_/MIC_90_ for teicoplanin, metronidazole, vancomycin and clindamycin to be 0.38/0.5, 0.38/0.5, 1/2 and 2/≥256 mg/L, respectively [4]. More recently, Ngamskulrungroj et al. also used E-strips to investigate 53 toxigenic *C. difficile* and reported the majority (98.2–100%) to be susceptible to metronidazole, vancomycin, tigecycline and daptomycin. High MICs (>32 mg/L) were observed in 100% and 43.4% of the strains tested against ciprofloxacin and moxifloxacin, respectively. The MIC_90_ of linezolid was 0.5 mg/L [5].

We recently investigated the epidemiology of CDI at Siriraj Hospital in Bangkok [6]. From 422 stool specimens, 100 (23.7%) grew *C. difficile* yielding 105 isolates. Interestingly, the majority of the strains (62.9%) did not contain genes encoding the major virulence factors toxin A or toxin B (A−B−), while 25.7% were A+B+ and 11.4% were A−B+. In contrast to much of the rest of the world, none of the isolates carried binary toxin genes (CDT−) [6]. The implications of a high prevalence of non-toxigenic strains are unknown. It is possible that non-toxigenic *C. difficile* could play a protective role against CDI in Thailand [7], or there may be other undetermined virulence factors in these lineages. Indeed, in the previously mentioned study [6] CDI cases exhibited lower morbidity and mortality rates compared to those seen in North America and Europe (Putsathit P. et al., unpublished data). Of concern is the fact that toxigenic strains are capable of converting toxin null strains to toxin producers via horizontal transfer of the pathogenicity locus [8] and that transfer of mobile genetic elements harbouring antimicrobial resistance (AMR) genes has been demonstrated in vitro [9]. Co-colonisation of toxigenic and non-toxigenic strains was demonstrated among Thai patients, indicating the possible risk of such conversion [6].

In the absence of contemporary data and continual injudicious use of antimicrobials in this region [2, 10], we investigated the antimicrobial susceptibility of these recently isolated strains of *C. difficile* in Thailand, including assessing the potential risk for AMR transfer from non-toxigenic strains.

## Methods

### Collection, isolation and characterisation of *C. difficile*

The 105 *C. difficile* strains isolated previously [6] belonged to 38 distinct PCR ribotypes (RTs), 55.2% (58/105) of which were assigned to internationally recognised RTs: 005 (*n* = 1), 009 (*n* = 6), 010 (*n* = 12), 014/020 group (*n* = 17), 017 (*n* = 12), 039 (*n* = 9) and 103 (*n* = 1). The remaining 44.8% (47/105) of the isolates did not match any reference strains and were designated with an internal nomenclature prefixed QX [6].

### Minimum inhibitory concentration (MIC) determination

In vitro susceptibility for fidaxomicin, vancomycin, metronidazole, rifaximin, clindamycin, erythromycin, amoxicillin/clavulanate, moxifloxacin and meropenem was performed by agar incorporation method. Testing and clinical breakpoint determination followed the recommended guidelines of Clinical and Laboratory Standards Institute (CLSI) and European Committee on Antimicrobial Susceptibility Testing (EUCAST) as previously described [11].

## Results

Summary MIC data for nine antimicrobials against *C. difficile* isolates are shown in Table 1. All isolates were susceptible to vancomycin, metronidazole, amoxicillin/clavulanate, and meropenem. The in vitro activity of fidaxomicin (MIC_50_/MIC_90_ 0.06/0.25 mg/L) was superior to vancomycin (MIC_50_/MIC_90_ 1/2 mg/L) and metronidazole (MIC_50_/MIC_90_ 0.25/0.25 mg/L). Compared to fidaxomicin, vancomycin and metronidazole, rifaximin exhibited a greater MIC range (0.008–>16 mg/L). The MIC of rifaximin was ≤0.03 mg/L against the majority of the isolates (85.7%; 90/105) and ≥16 mg/L for the remainder (14.3%; 15/105). The MIC_50_ of rifaximin (0.015 mg/L) was the lowest of all antimicrobials tested. Nearly 3/4 (73.3%; 77/105) of the isolates were resistant to clindamycin, while less resistance to erythromycin (35.2%; 37/105) and moxifloxacin (21.0%; 22/105) was observed. The prevalence of multi-drug resistance (MDR), as defined by resistance to ≥3 antimicrobials tested, was 21.9% (23/105). This included RTs 017 (*n =* 8), 039 (*n* = 6), QX002 (*n* = 3), 010 (*n* = 2), QX190 (*n* = 2), 009 (*n* = 1) and QX516 (*n* = 1). Except for RT 017, all of the MDR isolates were non-toxigenic (A−B−CDT−).

**Table 1:** Summary MIC data for nine antimicrobials against 105 Thai *C. difficile* isolates

Antimicrobial^a^	MIC range (mg/L)	MIC_50_ (mg/L)	MIC_90_ (mg/L)	Clinical breakpoints [11]			Susceptible		Intermediate		Resistant	
				S	I	R	N	%	N	%	N	%
FDX	0.004–0.25	0.06	0.25	–	–	–	–	–	–	–	–	–
VAN	0.06–2	1	2	≤2	–	>2	105	100.0	0	0.0	0	0.0
MTZ	0.015–0.5	0.25	0.25	≤2	–	>2	105	100.0	0	0.0	0	0.0
RFX	0.008–>16	0.015	>16	–	–	–	–	–	–	–	–	–
CLI	0.015–>32	8	>32	≤2	4	≥8	17	16.2	11	10.5	77	73.3
ERY	0.12–>256	2	>256	–	–	>8	–	–	–	–	37	35.2
AMC	0.03–2	0.5	1	≤4	8	≥16	105	100.0	0	0.0	0	0.0
MXF	0.12–32	2	16	≤2	4	≥8	82	78.1	1	1.0	22	21.0
MEM	0.25–4	2	4	≤4	8	≥16	105	100.0	0	0.0	0	0.0

^a^ *FDX* fidaxomicin, *VAN* vancomycin, *MTZ* metronidazole, *RFX* rifaximin, *CLI* clindamycin, *ERY* erythromycin, *AMC* amoxicillin/clavulanate, *MXF* moxifloxacin, *MEM* meropenem

Summary MIC and susceptibility data for the nine antimicrobials against *C. difficile* by toxin gene profiles are shown in Table 2. The MIC_50_/MIC_90_ values of rifaximin against non-toxigenic and A+B+ isolates were identical (0.015/0.03 mg/L) and lower than those of A−B+ isolates (>16/>16 mg/L), all of which were RT 017. Similar trends for clindamycin, erythromycin and moxifloxacin were observed where MICs for A−B+ isolates were higher than those of non-toxigenic and A+B+ isolates (Table 2). Comparable levels of resistance to clindamycin were observed for non-toxigenic (77.3%), A+B+ (66.7%) and A−B+ (66.7%) isolates. Compared to non-toxigenic isolates, relatively higher proportions of A−B+ isolates were resistant to erythromycin (40.9% vs. 83.3%) and moxifloxacin (18.2% vs. 83.3%). No erythromycin and moxifloxacin resistance was observed in A+B+ isolates.

**Table 2:** Summary MIC data for nine antimicrobials against Thai *C. difficile* isolates by toxin gene profile

Antimicrobial^a^	Toxigenic isolates (*n* = 39)		A−B− isolates (*n* = 66)		A+B+ isolates (*n* = 27)		A−B+ isolates (*n* = 12)	
	MIC Range	MIC_50_/MIC_90_	MIC Range	MIC_50_/MIC_90_	MIC Range	MIC_50_/MIC_90_	MIC Range	MIC_50_/MIC_90_
FDX	0.008–0.25	0.06/0.25	0.004–0.25	0.06/0.25	0.015–0.25	0.12/0.25	0.008–0.06	0.03/0.06
VAN	0.25–2	1/2	0.06–2	1/1	0.25–2	1/2	0.5–2	1/1
MTZ	0.015–0.25	0.25/0.25	0.015–0.5	0.25/0.5	0.015–0.25	0.25/0.25	0.015–0.25	0.12/0.25
RFX	0.015–>16	0.03/>16	0.008–>16	0.015/0.03	0.015–0.03	0.015/0.03	0.015–>16	>16/>16
CLI	0.015–>32	8/>32	0.03–>32	8/>32	0.015–16	8/16	0.015–>32	>32/>32
ERY	0.5–>256	2/>256	0.12–>256	2/>256	0.5–2	2/2	1–>256	>256/>256
AMC	0.03–1	0.5/1	0.03–2	0.5/1	0.03–1	0.5/1	0.03–1	0.5/1
MXF	0.12–>32	2/32	0.12–>32	2/16	0.12–4	2/2	0.25–32	16/32
MEM	0.25–4	2/4	0.25–4	2/4	0.5–4	2/4	0.25–4	4/4

^a^ *FDX* fidaxomicin, *VAN* vancomycin, *MTZ* metronidazole, *RFX* rifaximin, *CLI* clindamycin, *ERY* erythromycin, *AMC* amoxicillin/clavulanate, *MXF* moxifloxacin, *MEM* meropenem. All values are shown in mg/L

## Discussion

To investigate current trends in the antimicrobial susceptibility of Thai *C. difficile*, 105 recent isolates were tested against nine antimicrobials. Reduced susceptibility to metronidazole (MIC >2 mg/L) was not recorded, and the highest MIC/MIC_50_/MIC_90_ values (0.5/0.25/0.25 mg/L, respectively) were lower than those previously reported for Thailand [4]. The lack of resistance against metronidazole is consistent with studies conducted in Australia (MIC range 0.12–1 mg/L; *n* = 440 [11]) and North America [12], although reduced susceptibility was documented in other North American and European studies [13]. A recent study from Taiwan also reported reduced susceptibility to metronidazole (MIC range of ≤0.03–4 mg/L), however, growth of 90% of isolates (*n* = 403) was inhibited at 0.5 mg/L [14]. Metronidazole MICs of ≥4 mg/L were also observed in 5.3% (7/131) of *C. difficile* isolated in Korea [15].

The MIC_50_/MIC_90_ for vancomycin (1/2 mg/L) were identical to those reported in Thailand by Wongwanich et al., however, according to the epidemiological cut-off (ECOFF) value for vancomycin (>2 mg/L), their MIC range (0.5–3 mg/L) suggests reduced susceptibility [4]. A study conducted in North America reported a decrease in vancomycin susceptibility (MICs of 4 mg/L) in 13.2% (40/302) of isolates, including 39.1% (34/87) of RT 027 strains. None of the isolates carried *vanA* or *vanB* genes [12]. Reduced susceptibility (MIC 4 mg/L) and resistance (MIC >8 mg/L) to vancomycin was also observed in 2.3% and 0.9% of the European isolates (*n* = 918), respectively [16]. Vancomycin MICs of 4 mg/L were also reported in North America and Europe [13], Taiwan (2/403) [14] and Korea (4/131) [15]. With the appearance of *C. difficile* with reduced vancomycin susceptibility, the ‘vancomycin MIC creep’ observed among methicillin-resistant *Staphylococcus aureus* and the global dissemination of vancomycin resistant enterococci [17], appropriate and controlled use of this antimicrobial is increasingly important. However, with regards to CDI, the significance of such increases is doubtful given the high faecal levels of vancomycin achieved with the standard treatment regime (125 mg orally four times daily) [13].

In the current study, the in vitro activity of fidaxomicin was superior to vancomycin and metronidazole. Using the ECOFF value recommended by the European Medicines Agency (MIC of >1 mg/L) [18], no resistance was observed. The lack of resistance is consistent with previous studies conducted in Europe (MIC_50_/MIC_90_ 0.06/0.25 mg/L; *n* = 918) [16], North America and Europe (MIC_50_/MIC_90_ 0.125/0.25 mg/L; *n* = 719) [13], Australia (MIC_50_/MIC_90_ 0.03/0.12 mg/L; *n* = 440) [11] and Taiwan (MIC_50_/MIC_90_ 0.12/0.25 mg/L; *n* = 403) [14]. A disadvantage of using fidaxomicin in developing countries is the high cost although a European study suggested that by providing this agent to a targeted patient population in whom superior outcome has been demonstrated, the cost could be indirectly reduced through the shorter period of hospitalisation [19]. The relevance of this in Asia is unclear.

Rifaximin is still under evaluation for treatment of CDI. Although highly active against the majority of *C. difficile* tested, some strains had high MICs [13, 16]. These observations are consistent with results obtained here, where 85.7% of the isolates had MICs of ≤0.03 mg/L. Isolates reported to have high MICs included RT 027, which harboured a mutation in the gene encoding RNA polymerase B (*rpoB*) [20]. In our study rifaximin MIC_50_/MIC_90_ values for RT 017 (A−B+) isolates (>16/>16 mg/L) were higher than those of non-toxigenic and A + B+ isolates (0.015/0.03 mg/L in both groups). This suggests a possible alteration in *rpoB* and warrants further investigation. Rifaximin resistance was previously defined as an MIC of ≥32 mg/L [20]. The highest concentration of rifaximin investigated in this study was 16 mg/L and 13.3% of isolates had MICs of >16 mg/L, indicating possible resistance.

Fluoroquinolones have been strongly associated with CDI and linked to the large outbreaks in Quebec hospitals [21]. In 2011, a study was conducted in a large tertiary hospital in Bangkok to investigate the antimicrobial prescription patterns for adults with acute diarrhoea. Overall, inappropriate use of antimicrobials was 48.9%, with fluoroquinolones and the third generation cephalosporin ceftriaxone being the top agents prescribed [10]. It is therefore not surprising that resistance to moxifloxacin (MIC ≥8 mg/L) was observed in 21.0% of Thai isolates given the rapid rate that resistance to quinolones develops following exposure [22]. In Thailand, fluoroquinolone resistance was particularly pronounced among A−B+, RT 017 isolates (83.3%), compared to non-toxigenic (18.2%) and A+B+ (0.0%) isolates. High proportions of fluoroquinolone resistance were also observed in Taiwan (17.9%; *n =* 403) [14], North America (35.8%) [12], South Korea (62.6%; *n =* 131) [15], and Europe (40.0% to moxifloxacin; *n =* 918 [16]). In contrast, limited resistance has been observed in Australia (3.4%; *n =* 440 [11]), likely due to Australia’s strict national guidelines for quinolones use in human and their prohibition of use in production animals [23].

A Korean study reported a correlation between the use of clindamycin and the incidence of CDI [24]. In Asia, *C. difficile* RT 017 is highly prevalent and is known to harbour the *ermB* gene [15]. This observation supports the high level of resistance to clindamycin (73.3%) and erythromycin (35.2%) observed among Thai isolates, particularly in RT 017 (66.7% and 83.3%, respectively). High proportions of clindamycin resistance (MIC of ≥8 mg/L) were also observed in Europe (50%; *n =* 917 [16]) and North America (36.8%) [12]. The *ermB* gene encodes a 23S rRNA methylase conferring resistance to lincosamides and macrolides and is carried on transposons (e.g. Tn*6215*, Tn*6218,* Tn*6194* and Tn*5398*, the latter notably having two copies) [25]. CDI caused by strains carrying Tn*6194* was more severe with greater mortality (29% vs. 3%) [26]. Tn*6194* is the most common *ermB*-containing element in European clinical isolates [27] and carries genes for recombinases and integrases which enable intra- and inter-species (*Enterococcus faecalis*) transfer [28].

In this study, MDR was prevalent (21.9%) in both toxigenic and non-toxigenic strains. Of note, 66.7% of RT 017 strains showed multiple resistance. Similar results were observed among RT 017 strains in a North American study that tested 508 *C. difficile* against six antimicrobials (metronidazole, vancomycin, rifampin, clindamycin, moxifloxacin and tetracycline) [12]. Apart from RT 017, the rest of the MDR strains were non-toxigenic; thus, the previously demonstrated intra-species transfer of the pathogenicity locus [8] and AMR genes [9] suggests that these MDR non-toxigenic strains of *C. difficile* are an important consideration.

## Conclusions

*C. difficile* in Thailand is characterised by a high level of MDR. This includes resistance to fluoroquinolones which are frequently used to treat acute diarrhoeal disease. Education plays a pivotal role in creating behavioural changes and healthcare professionals should be encouraged to inform their patients about the importance of antibiotic stewardship. Although toxigenic culture is not popular as a standalone diagnostic test, stool culturing should still be performed to enable surveillance of the ever-changing epidemiology of CDI and, in particular, the development of AMR. This study demonstrates that both non-toxigenic *C. difficile* and RT 017 strains can be a significant reservoir of AMR and is yet another reminder of the urgent need for antibiotic stewardship in Asia in general and Thailand specifically.

## Abbreviations

A+/−: Toxin A gene positive/negative
AMC: Amoxicillin/clavulanate
AMR: Antimicrobial resistance
B+/−: Toxin B gene positive/negative
CDI: *Clostridium difficile* infection
CDT+/−: Binary toxin gene positive/negative
CLI: Clindamycin
CLSI: Clinical and Laboratory Standards Institute
ECOFF: Epidemiological cut-off MIC value which distinguish wild-type isolates from those with reduced susceptibility
ERY: Erythromycin
EUCAST: European Committee on Antimicrobial Susceptibility Testing
FDX: Fidaxomicin
MDR: Multi-drug resistance
MEM: Meropenem
mg/L: Milligrams per liter
MIC: Minimum inhibitory concentration
MIC_50_: MIC required to inhibit the growth of 50% of organisms
MIC_90_: MIC required to inhibit the growth of 90% of organisms
MTZ: Metronidazole
MXF: Moxifloxacin
nm: Nanometer
PCR: Polymerase chain reaction
QX: Internal nomenclature, prefix for novel PCR ribotypes
RFX: Rifaximin
RT: PCR ribotype
VAN: Vancomycin
WHO: World Health Organisation

## References

[1] Bignardi GE (1998). Risk factors for *Clostridium difficile* infection. J Hosp Infect.

[2] Sumpradit N, Chongtrakul P, Anuwong K, Pumtong S, Kongsomboon K, Butdeemee P (2012). Antibiotics Smart Use: a workable model for promoting the rational use of medicines in Thailand. Bull World Health Organ.

[3] Kusum M, Wongwanich S (1994). Susceptibility of *Clostridium difficile* to sixteen antimicrobial agents. J Health Sci.

[4] Wongwanich S, Kusum M, Phan-Urai R (1996). Antibacterial activity of teicoplanin against *Clostridium difficile*. Southeast Asian J Trop Med Public Health.

[5] Ngamskulrungroj P, Sanmee S, Putsathit P, Piewngam P, Elliott B, Riley TV (2015). Molecular epidemiology of *Clostridium difficile* infection in a large teaching hospital in Thailand. PLoS One.

[6] Putsathit P, Maneerattanaporn M, Piewngam P, Kiratisin P, Riley T (2017). Prevalence and molecular epidemiology of *Clostridium difficile* infection in Thailand. New Microbes New Infect.

[7] Gerding DN, Meyer T, Lee C, Cohen SH, Murthy UK, Poirier A (2015). Administration of spores of nontoxigenic *Clostridium difficile* strain M3 for prevention of recurrent *C. difficile* infection: a randomized clinical trial. JAMA.

[8] Brouwer MS, Roberts AP, Hussain H, Williams RJ, Allan E, Mullany P (2013). Horizontal gene transfer converts non-toxigenic *Clostridium difficile strains* into toxin producers. Nat Commun.

[9] Goh S, Hussain H, Chang BJ, Emmett W, Riley TV, Mullany P. Phage C2 mediates transduction of Tn6215, encoding erythromycin resistance, between *Clostridium difficile* strains. MBio. 2013;4(6):e00840–13. 10.1128/mBio.00840-13 PMC3870246 24255122

[10] Supcharassaeng S, Suankratay C (2011). Antibiotic prescription for adults with acute diarrhea at King Chulalongkorn Memorial Hospital, Thailand. J Med Assoc Thai.

[11] Knight DR, Giglio S, Huntington PG, Korman TM, Kotsanas D, Moore CV (2015). Surveillance for antimicrobial resistance in Australian isolates of *Clostridium difficile*, 2013–14. J Antimicrob Chemother.

[12] Tickler IA, Goering RV, Whitmore JD, Lynn AN, Persing DH, Tenover FC (2014). Strain types and antimicrobial resistance patterns of *Clostridium difficile* isolates from the United States, 2011 to 2013. Antimicrob Agents Chemother.

[13] Goldstein EJ, Citron DM, Sears P, Babakhani F, Sambol SP, Gerding DN (2011). Comparative susceptibilities to fidaxomicin (OPT-80) of isolates collected at baseline, recurrence, and failure from patients in two phase III trials of fidaxomicin against *Clostridium difficile* infection. Antimicrob Agents Chemother.

[14] Liao CH, Ko WC, Lu JJ, Hsueh PR (2012). Characterizations of clinical isolates of *Clostridium difficile* by toxin genotypes and by susceptibility to 12 antimicrobial agents, including fidaxomicin (OPT-80) and rifaximin: a multicenter study in Taiwan. Antimicrob Agents Chemother.

[15] Kim J, Kang JO, Pai H, Choi TY (2012). Association between PCR ribotypes and antimicrobial susceptibility among *Clostridium difficile* isolates from healthcare-associated infections in South Korea. Int J Antimicrob Agents.

[16] Freeman J, Vernon J, Morris K, Nicholson S, Todhunter S, Longshaw C (2015). Pan-European longitudinal surveillance of antibiotic resistance among prevalent *Clostridium difficile* ribotypes. Clin Microbiol Infect.

[17] Willems R, Top J, Van Santen M, Robinson DA, Coque TM, Baquero F (2005). Global spread of vancomycin-resistant *Enterococcus faecium* from distinct nosocomial genetic complex. Emerg Infect Dis.

[18] Committee for Medicinal Products for Human Use (CHMP) EMA. Assessment report - Fidaxomicin (Procedure No: EMEA/H/C/2087). 2011.

[19] Rubio-Terres C, Cobo Reinoso J, Grau Cerrato S, Mensa Pueyo J, Salavert Lleti M, Toledo A (2015). Economic assessment of fidaxomicin for the treatment of *Clostridium difficile* infection (CDI) in special populations (patients with cancer, concomitant antibiotic treatment or renal impairment) in Spain. Eur J Clin Microbiol Infect Dis.

[20] O’Connor JR, Galang MA, Sambol SP, Hecht DW, Vedantam G, Gerding DN (2008). Rifampin and rifaximin resistance in clinical isolates of *Clostridium difficile*. Antimicrob Agents Chemother.

[21] Pepin J, Saheb N, Coulombe MA, Alary ME, Corriveau MP, Authier S (2005). Emergence of fluoroquinolones as the predominant risk factor for *Clostridium difficile*-associated diarrhea: a cohort study during an epidemic in Quebec. Clin Infect Dis.

[22] Ackermann G, Tang-Feldman YJ, Schaumann R, Henderson JP, Rodloff AC, Silva J (2003). Antecedent use of fluoroquinolones is associated with resistance to moxifloxacin in *Clostridium difficile*. Clin Microbiol Infect.

[23] Cheng AC, Turnidge J, Collignon P, Looke D, Barton M, Gottlieb T (2012). Control of fluoroquinolone resistance through successful regulation, Australia. Emerg Infect Dis.

[24] Kim J, Kang JO, Kim H, Seo MR, Choi TY, Pai H (2013). Epidemiology of *Clostridium difficile* infections in a tertiary-care hospital in Korea. Clin Microbiol Infect.

[25] Leclercq R (2002). Mechanisms of resistance to macrolides and lincosamides: nature of the resistance elements and their clinical implications. Clin Infect Dis.

[26] Corver J, Bakker D, Brouwer MS, Harmanus C, Hensgens MP, Roberts AP (2012). Analysis of a *Clostridium difficile* PCR ribotype 078 100 kilobase island reveals the presence of a novel transposon, Tn6164. BMC Microbiol.

[27] Spigaglia P (2016). Recent advances in the understanding of antibiotic resistance in *Clostridium difficile* infection. Ther Adv Infect Dis.

[28] Knight DR, Elliott B, Chang BJ, Perkins TT, Riley TV (2015). Diversity and evolution in the genome of *Clostridium difficile*. Clin Microbiol Rev.

